# Case report: Staged tension-reducing excision of giant acquired vulvar lymphangioma secondary to cervical cancer surgery

**DOI:** 10.3389/fonc.2024.1418829

**Published:** 2024-09-06

**Authors:** Ling-Juan Hu, Hao-Ming Fang, Huan-Mei Lin, Xu Kang, Ying Lin, Jing Xiao

**Affiliations:** ^1^ The Second Clinical College of Guangzhou University of Chinese Medicine, Guangzhou, Guangdong, China; ^2^ Department of Gynecology, The Second Affiliated Hospital of Guangzhou University of Chinese Medicine, Guangzhou, Guangdong, China; ^3^ Clinical Division, School of Chinese Medicine, Hong Kong Baptist University, Kowloon, Hong Kong SAR, China; ^4^ Department of Dermatology, The Second Affiliated Hospital of Guangzhou University of Chinese Medicine, Guangzhou, Guangdong, China; ^5^ State Key Laboratory of Traditional Chinese Medicine Syndrome/Department of Gynecology, The Second Affiliated Hospital of Guangzhou University of Chinese Medicine, Guangzhou, Guangdong, China

**Keywords:** staged excision, cervical cancer, surgery, case report, vulvar lymphangioma

## Abstract

**Introduction:**

Acquired vulvar lymphangioma (AVL), a rare disease caused by the dilation of superficial lymphatic vessels secondary to deep lymphatic vessel injury, is characterized by a wide range of morphological diversity and massive exudate. This morphological heterogeneity has often led to misdiagnosis or non-diagnosis. The management of AVL presents a therapeutic challenge due to the absence of a standardized treatment protocol.

**Case presentation:**

A 53-year-old female patient, previously received surgical treatments for stage IIb cervical squamous cell carcinoma, presented with vulvar enlargement and copious amount of yellow exudate seven years post-treatment. Clinically, the patient exhibited chronic vulvar swelling, with easily-exudated nodules. The vulvar biopsy revealed lymphatic vessel dilation with lymphocyte infiltration, consistent with AVL. Due to the extensive lesions and severe exudate, staged excisions of bilateral vulvar lesions were performed at one-month intervals. Follow-up examinations of this patient for one-year post-surgery showed no evidence of recurrence.

**Conclusion:**

In this instance, AVL manifest secondary to cervical cancer surgery, as a result of damage to the deep lymphatic vessels of the vulva, with characteristic symptoms of copious amounts of exudate and vulvar lesions with diverse morphologies, which provides a cautionary note for physicians. Besides, the staged resection strategy in this case may offer insights into surgical treatment protocol for extensive AVL.

## Introduction

AVL, also known as secondary lymphangiectasia, is a rare condition resulting from the dilation of superficial lymphatic vessels after injury to normal deep lymphatic vessel (1, 2). The etiology of lymphangiomas may include surgery, trauma, infection, tumor radiotherapy, or chronic non-specific inflammation, which can damage the normal pelvic lymphatic vessels, leading to vulvar lymphatic drainage disorders and lymphatic vessel dilation (3, 14, 16). Given the absence of established treatment protocols for AVL, this report details the case of a patient who developed AVL following surgical and adjuvant interventions for cervical cancer. The patient’s condition was notable for its wide morphological diversity in lesions and significant exudative discharge. Consequently, a staged tension-reducing surgical approach was employed to excise the lesions.

## Case presentation

We present the case of a 53-year-old female diagnosed with stage IIb cervical squamous cell carcinoma in 2009. She underwent surgical intervention and received adjuvant radiotherapy and chemotherapy. Subsequent follow-up examinations revealed no evidence of recurrence. However, five years post-treatment, the patient developed vulvar enlargement and exudate production, without associated pain or pruritus. Over the ensuing seven years, her symptoms progressively aggravated; the volume of yellow exudate increased steadily, to the extent that it could saturate an overnight sanitary napkin within an hour. Despite pharmacological management, her condition deteriorated, necessitating the use of diapers continuously. These persistent and deteriorating symptoms had markedly diminished her quality of life, compelling her to seek further medical intervention.

**Figure 1:**
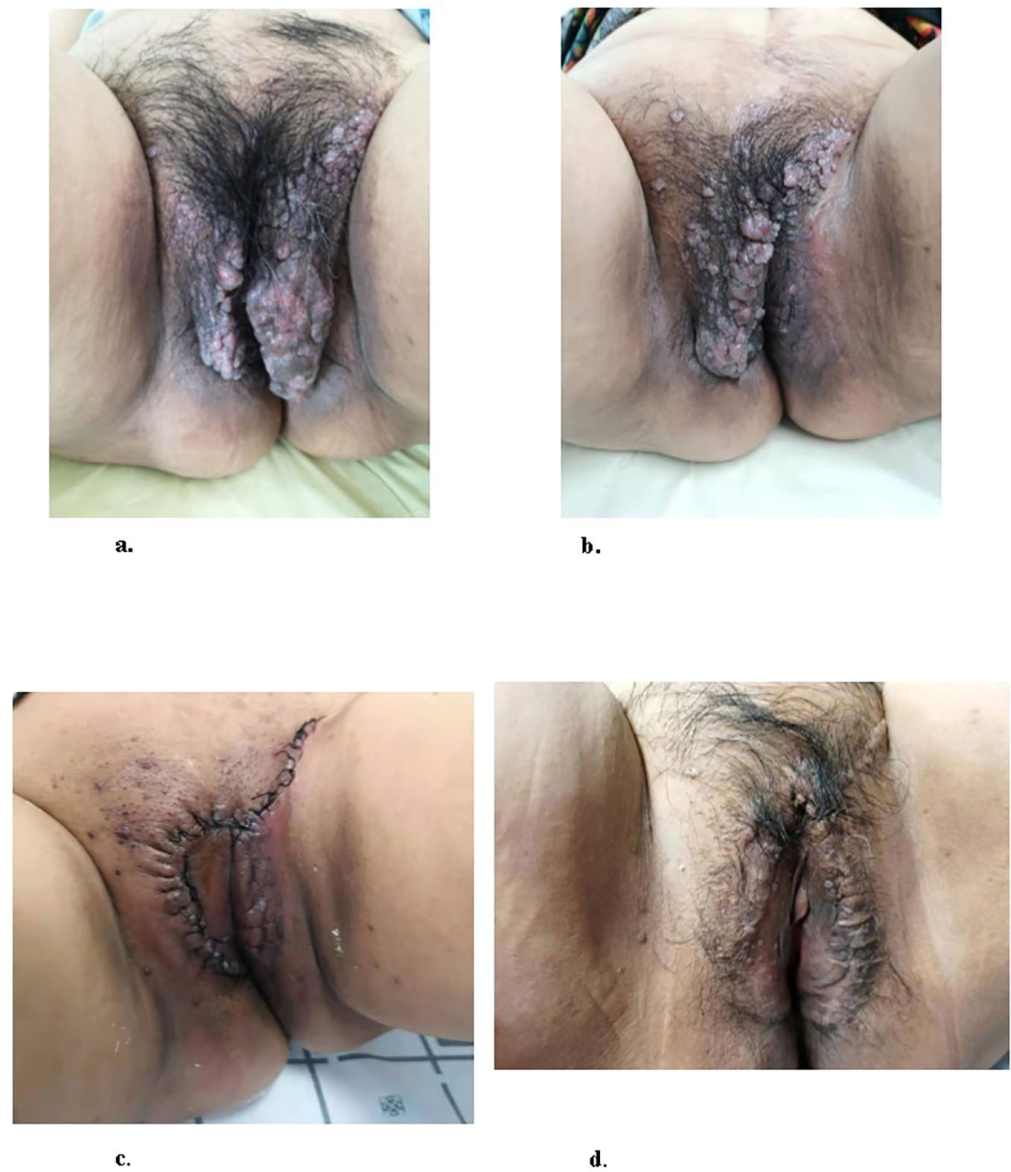
Images of the patient vulva at different stage. **(A)** Physical examination of the patient prior to surgery demonstrated the presence of vulvar swelling. **(B)** Vulva of the patient, one month after the first resection of the lesion on the left labium minus. **(C)**. Image of the patient vulva, post-second resection of the lesions on the left labium majus and the right side of mons pubis. **(D)**. Follow-up examination for 1-year post-surgery showed no obvious signs of recurrence.

**Figure 1A** illustrated the results of the patient’s physical examination, which revealed chronic vulvar swelling, involving both labia majora. The swelling was notably more pronounced on the left side, measuring 25×15 cm. The skin surface exhibited multiple lesions, including papules, papulovesicles, and nodules, the majority of which were densely clustered like frog eggs, while a minority were scattered and verrucous. These lesions varied in color from skin-toned to dark red, and presented with diverse textures ranging from smooth to erosive. Upon palpation, these lesions were cystic, tender, and easy to exudate. Subsequent vulvar biopsy confirmed the diagnosis of AVL, characterized by chronic inflammation of the skin tissue and cystic expansion.

**Figure 2:**
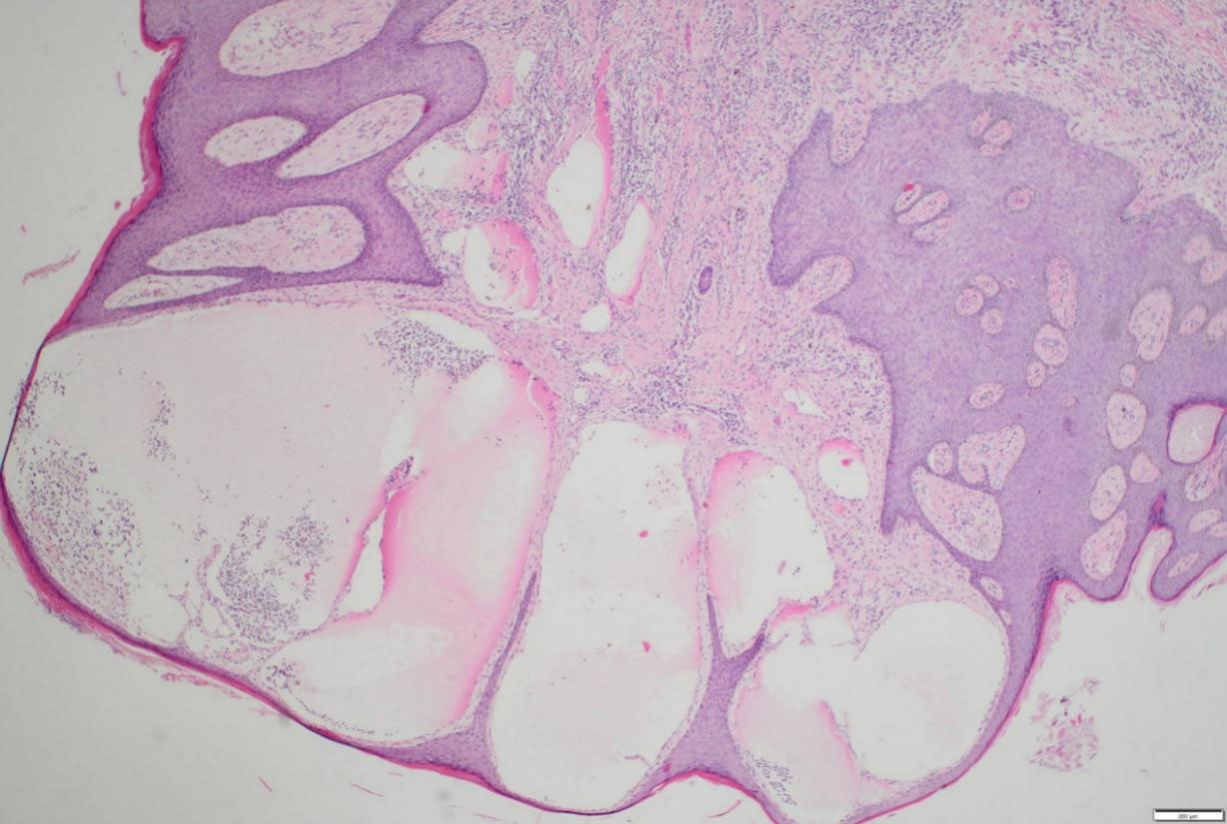
Multiple dilated lymphatic vessels, with differing lumen sizes and irregular shapes, are present in superficial and deep dermis of a vulvar lesion tissue section, as shown by hematoxylin & eosin (H&E) staining.

**Figure 3:**
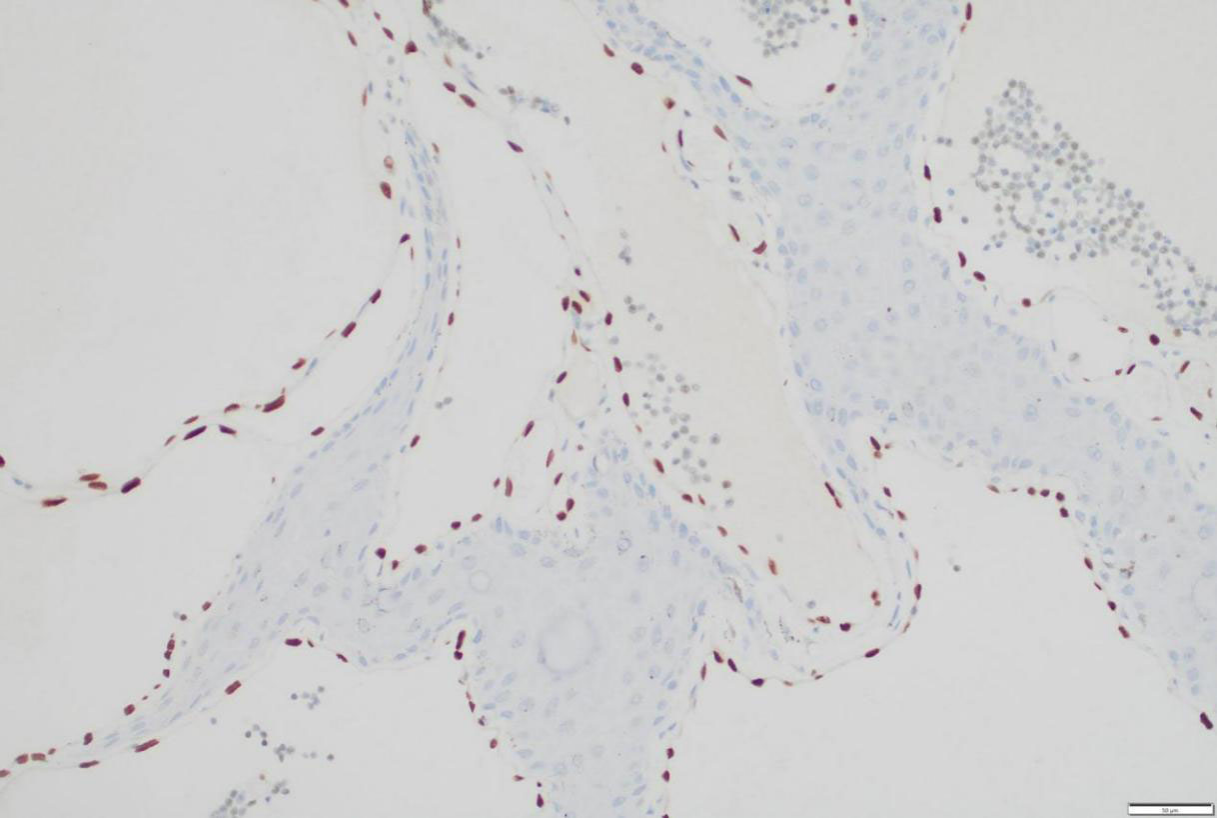
Immunohistochemical staining for D2-40, a marker for lymphatic endothelial cells, within the resected vulvar lesion.

**Figure 4:**
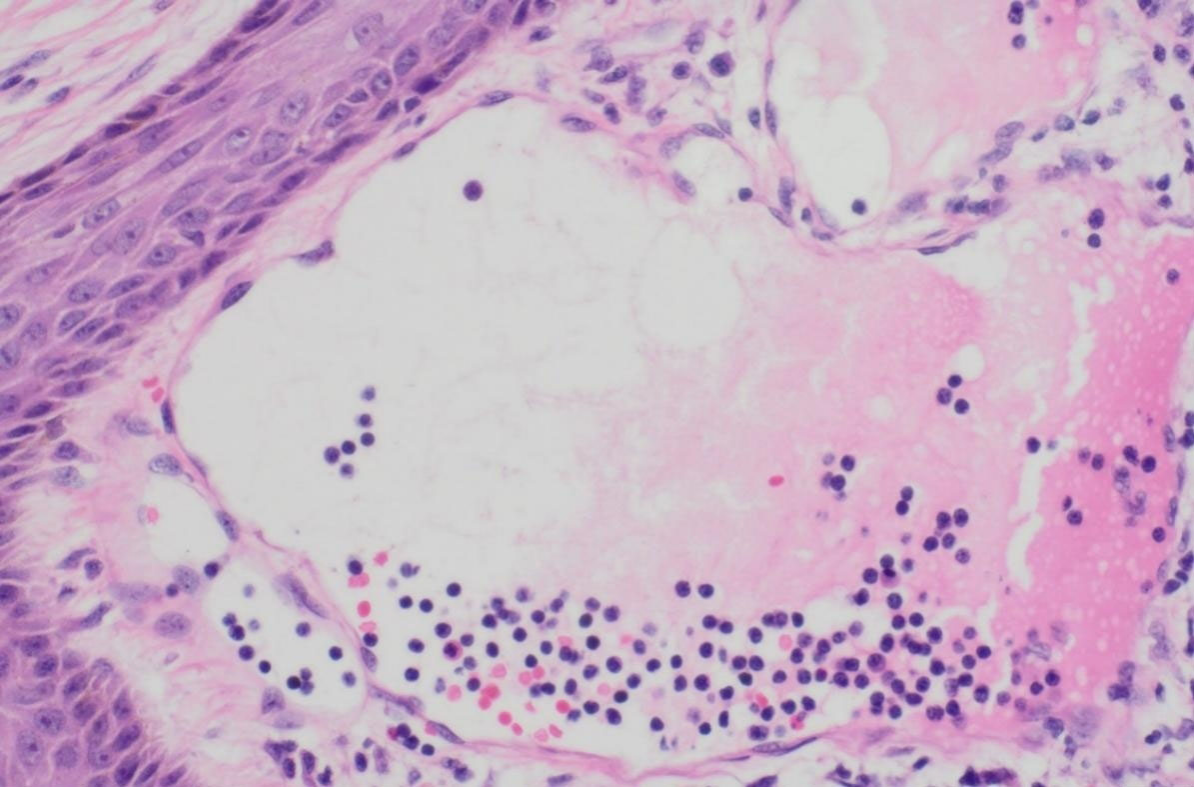
Lumen of dilated lymphatic vessels within the resected vulvar lesion, depicting the presence of a substantial infiltration of plasma cells and lymphocytes, under H&E staining.

**Figure 5:**
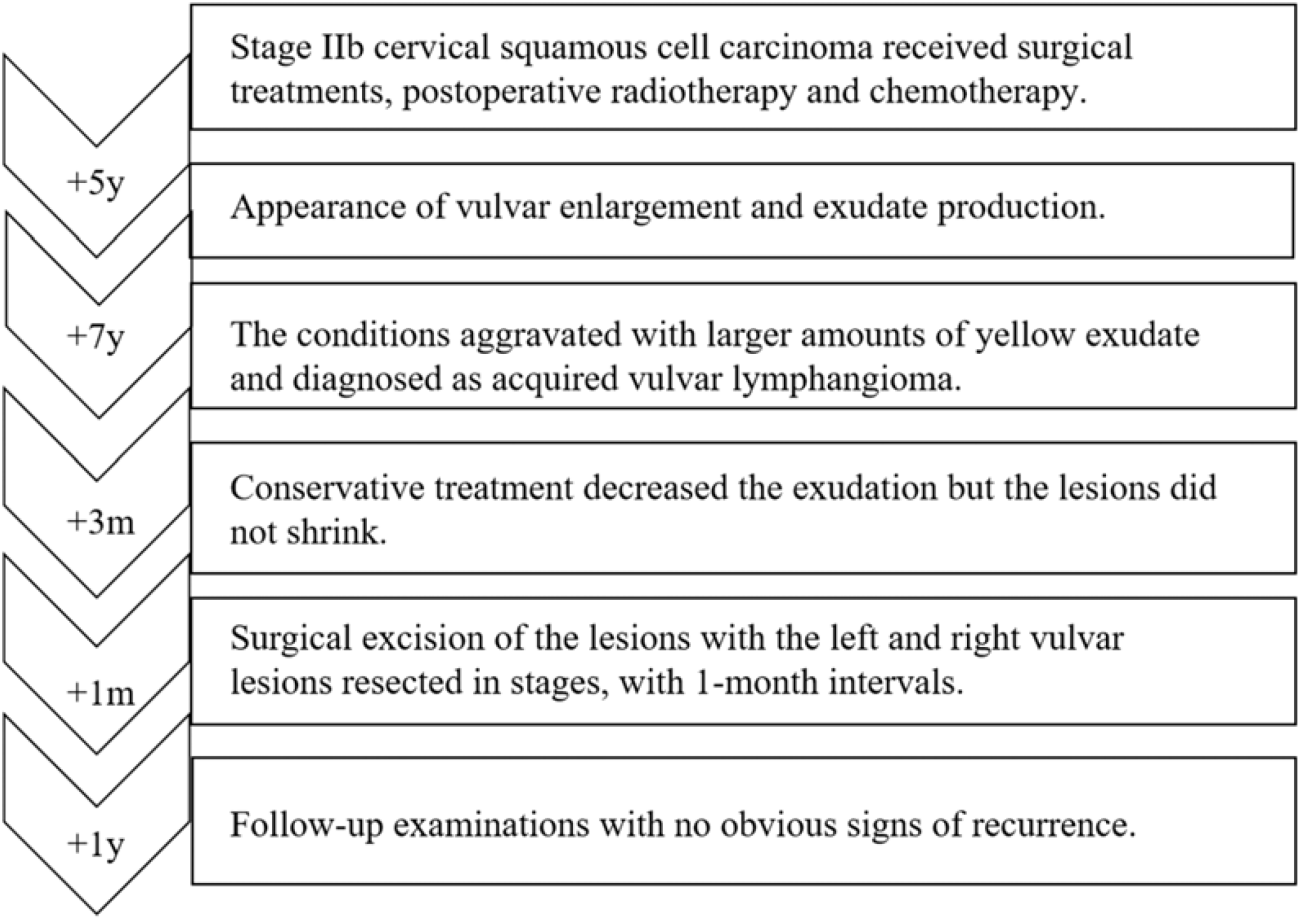
Historical and current information from this episode of care organized as a imeline.

In consideration of the wide range of lesions and severe exudate, the patient was given conservative treatment to decrease the exudation, but the lesions did not shrink. Owing to the size of the lesions and physical friction, the exudate remained uncontrolled after the cessation of pharmacotherapy. In order to mitigate the exudate and excise the lesions, surgical resection was pursued, which represented the predominant treatment approach for AVL (4, 13, 15). The surgical planning focused on achieving complete lesion removal while ensuring optimal incision healing. A single-stage excision might present considerable risks, including pronounced surgical trauma and significant wound tension, which were not conducive to postoperative tissue repair. Therefore, an individualized treatment regimen was employed. Firstly, based on the morphology of the lesions, the incision shape was designed like , to facilitate low-tension wound closure after lesion excision. Additionally, a staged surgical approach was adopted to mitigate surgical trauma-related complications. The initial excision involved the lesion on the left labium minus (illustrated as the green area in the graph above), followed by a second excision after a one-month interval, encompassing the lesions on the left labium majus (indicated by the red area in the diagram) and the right side of the mons pubis, as shown in **Figures 1B, C**. Pathological examination of the excised specimen revealed multiple dilated lymphatic vessels of varying lumen sizes and irregular shapes in superficial and deep dermis, as illustrated in **Figure 2**. The lymphatic vessels were delineated by D2-40 staining, a specific marker for lymphatic endothelial cells, as shown in **Figure 3**. Concomitant findings of substantial infiltration of plasma cells and lymphocytes around the dermal vascular plexus also corroborate the diagnosis of AVL (lymphangiectasia), as indicated in **Figure 4**. Follow-up examinations for 1-year post-surgery showed no obvious signs of recurrence, as shown in **Figure 1D**. A chronological overview of the complete case is presented on a timeline in **Figure 5**.

## Discussion

The presence of extensive lesions and massive exudate in this patient could be attributable to several factors, one of which is the unique anatomical characteristics of the vulva. A network of lymphatic capillaries is located in the superficial dermis or lamina propria. Specifically, the lymphatic capillaries within the labia majora, labia minora, clitoris, adjacent perineum and lower vaginal segment are likely anastomosed, forming an extensive network (5). Additionally, the capillary lymphatic networks on both sides of the labia are interconnected. Any hindrance to the lymphatic drainage can impact extensive portions of the vulva, yielding large lesions vulnerable to external exposure and resulting in the production of significant exudate.

Another potential contributing factor is the impact of surgical injuries on the return flow of lymphatic fluid within the vulva. The vulvar lymphatic capillaries, which connect to the deep lymphatic vessels located in the superficial layer of the subcutaneous tissue, play a crucial role in draining lymphatic fluid to the celiac lymph nodes (5). These lymph nodes are often excised during surgical treatments for cervical cancer (6). Consequently, the surgical damage to the deep lymphatic vessels can lead to vulvar lymphatic drainage disorder, impeding the efficient lymphatic reflow into the systemic circulation (7). The continuous production and retention of lymph fluid cause the dilation of vulvar capillary lymphatic vessels, progressively resulting in vulvar enlargement (4). The resultant enlargement of the vulva exacerbates friction against the skin and clothing, leading to skin damage and exudation of lymphatic fluid. Moreover, the obstruction of lymph flow and elevated pressure in the vulvar lymphatic vessels further aggravate this condition. Therefore, this case highlights a possible pathological complication associated with cervical cancer surgery, underscoring the need for heightened awareness among physicians regarding such postoperative consequences.

AVL presents a therapeutic challenge as there is no standard treatment protocol. Depending on the type and extent of the lesions, treatment options include sclerotherapy, electrocoagulation, cryotherapy with liquid nitrogen, carbon dioxide laser therapy, radiotherapy and surgical resection, which is commonly indicated for resection of extensive lesions (2, 8–13). The staged excision strategy in this case may serve as an exploration of operative treatment protocol for extensive AVL, allowing for incremental removal of the lesion while minimizing the risk of complications associated with large excisions. Moreover, addressing the underlying etiology is essential for enhancing the efficacy of management strategies for AVL, which includes eradication of contributing factors, protection or reestablishment of normal lymphatic circulation. Besides, the long-term effect of surgical resection still requires follow-up evaluation. The refinement of surgical strategies for extensive AVL necessitates further investigation.

## Patient perspective

“For the past 7 years, I have endured severe pain. Thanks to the surgical intervention of my doctor, my issue was successfully resolved, freeing me from the need to wear diapers, and enhancing my life with greater comfort and convenience.”

## Data Availability

The raw data supporting the conclusions of this article will be made available by the authors, without undue reservation.
